# Conservative management of a large idiopathic pulmonary artery aneurysm: A case report

**DOI:** 10.1016/j.amsu.2022.103853

**Published:** 2022-05-29

**Authors:** Morgan Turnow, Sidney Elston, Hafez Golzarian, Sreenivasa Chanamolu

**Affiliations:** a University of Pikeville Kentucky College of Osteopathic Medicine, Pikeville, KY, USA; b Mercy Health St. Rita's Medical Center, Internal Medicine Residency, Lima, OH, USA; c Mercy Health Pulmonary Disease Center, St. Rita's Medical Center, Lima, OH, USA

**Keywords:** Aneurysm, Pulmonary artery aneurysm, Pulmonary artery, Idiopathic, Case report

## Abstract

**Introduction and importance:**

Pulmonary artery aneurysms are rare anomalies of the pulmonary vasculature. They are often asymptomatic and frequently an incidental finding on imaging or autopsy. It is imperative to closely monitor pulmonary artery aneurysms as they can result in sudden dissection, rupture, and death. Due to the rarity of this disease, the number of studies on pulmonary artery aneurysm management are limited and debated in the literature.

**Case presentation:**

We report a case of an initially symptomatic patient with dyspnea on exertion with an incidental finding of a large 5.0 × 6.4 cm pulmonary artery aneurysm that responded well to conservative management. Her dyspnea self-resolved and the decision was made to closely monitor the patient every three months with serial computed tomography angiography imaging.

**Clinical discussion:**

Idiopathic aneurysms of the main pulmonary artery are rare with a poorly understood pathogenesis primarily due to the limited number of cases. There are no clear guidelines for management, but the least invasive approach should be used due to the risk of serious adverse events. Pharmacologic treatment of underlying comorbidities and serial computed tomography angiography imaging should be considered as conservative management.

**Conclusion:**

Six months later, she remains hemodynamically stable and the aneurysm has decreased in size by 15%. This case highlights that conservative management should be considered first line therapy in asymptomatic, hemodynamically stable patients regardless of aneurysm size.

## Highlights

• Management of pulmonary artery aneurysms is debated in the literature regarding conservative vs. surgical management.
• Conservative management can lead to shrinkage of the pulmonary artery aneurysm.
• Patients without risk factors can be successfully managed by serial CTA imaging.

## Abbreviations

Pulmonary Artery Aneurysm: (PAA)
Computed Tomography Angiography: (CTA)
Respiratory syncytial virus: (RSV)

## 1 Introduction

A pulmonary artery aneurysm (PAA) is a rare condition of the pulmonary vasculature. They can arise from several etiologies including congenital, idiopathic, autoimmune, infectious, inflammatory, and malignant [1]. Patients often present with nonspecific symptoms ranging from an incidental finding on imaging to massive hemoptysis [2,3]. The most common presenting symptoms are dyspnea, pleuritic chest pain, and cough [3]. Diagnosis and treatment are critical due to the risk of enlargement and subsequent rupture. Rupture of PAA is associated with a nearly 50% mortality rate [4]. The mean pulmonary artery diameter is 25mm ± 3 mm with the upper limit of normal being 29 mm in males and 27 mm in females [5]. PAA is most accepted to be a dilation of at least 43 mm in males and 40 mm in females [6]. Guidelines for the screening, diagnosis, and management of this condition are not well defined, and management is variable depending on the underlying cause. We present a case of a 62-year-old female who presented to the emergency department with dyspnea and upon computed tomography angiography (CTA) imaging, a 5.0 × 6.4 cm pulmonary artery aneurysm was discovered. This case has been reported in line with the SCARE 2020 criteria [7].

## 2 Case Presentation

**Fig. 1:**
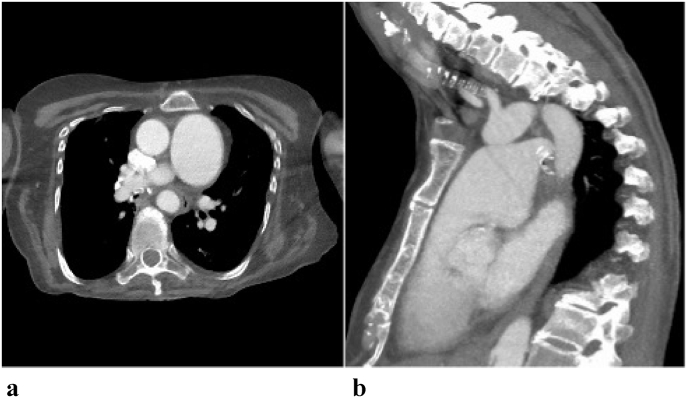
a (left): 1 mm axial view of CTA of the chest with and without contrast taken on initial presentation showing an aneurysmal dilatation of the main pulmonary artery measuring 5.0 × 6.4 cm. Patient was symptomatic with dyspnea on exertion at this time. Fig. 1b (right): Lateral view of initial CTA.

A 62-year-old female with a past medical history of anxiety, fibromyalgia, coronary artery disease, major depressive disorder, essential hypertension, and tobacco use presented to the emergency department via ambulance from her home with the chief complaint of shortness of breath that started several hours prior. She had no pertinent family history and she smoked half a pack of cigarettes daily. She had no known documented genetic history. Her home medications consisted of fluoxetine, lorazepam, tizanidine, amlodipine, aspirin, and atorvastatin. Her vital signs on admission revealed a blood pressure of 136/76 mmHg, heart rate 116 beats per minute, respiratory rate 16 per minute, temperature 98.5° Fahrenheit, 92% oxygen saturation on room air, and a BMI of 21.46. Arterial blood gas data revealed a pH 7.50, bicarbonate 33 mEq/L, and pCO_2_ 70 mmHg. Her D-Dimer was elevated at 900 ng/ml. She was placed on nasal cannula at a rate of 3.0 L/min due to increased oxygen demand. She only required room air at baseline. She was an active smoker with a 13-pack year smoking history but had quit 3 years prior. She had recently been hospitalized for a respiratory syncytial virus (RSV) infection one month prior. At this time, our differential diagnoses included pulmonary embolism, pneumonia, a new chronic obstructive pulmonary disease (COPD) exacerbation, and panic attack. On physical exam, her lungs were clear to auscultation bilaterally, no wheezing or rhonchi, and she had no cough. Thus, COPD and pneumonia were unlikely. A panic attack was also unlikely as her anxiety was well controlled for years with no identifiable trigger. Initial chest radiograph revealed mild cardiomegaly, and mild thickening of the interstitial lung markings but was overall unremarkable. A subsequent CTA of the chest revealed an intraluminal filling defect in the right bronchus intermedius, potentially representing mucous plugging or neoplasia. No embolism was seen on imaging. However, there was a large dilation of the main pulmonary artery measuring 5.0 × 6.4 cm in transverse and anteroposterior dimensions (Fig. 1a, Fig. 1b).

**Fig. 2:**
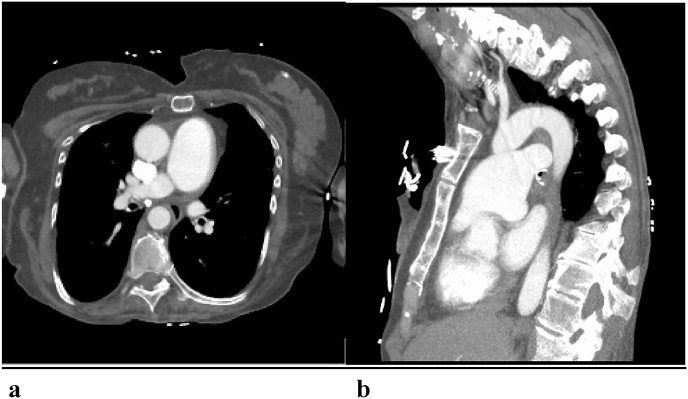
a (left): Repeat serial CTA of the chest at 6-month follow-up demonstrating a 15% reduction in size of the PAA. Fig. 2b: (right)**:** Lateral view of repeat CT Chest at 6-month follow-up demonstrating a 15% reduction in size of the PAA.

For the intraluminal filling defect, a bronchoscopy was initially planned. However, our patient's dyspnea spontaneously resolved by the following morning and on repeat CTA imaging, resolution of the defect was seen, leading us to believe that it was a mucous plug rather than neoplasm. Her bronchoscopy was subsequently canceled. After a thorough conversation with the patient, the decision was made by our cardiothoracic surgery team not to operate, as we felt the risks of the procedure outweighed the benefits given that she remained asymptomatic and hemodynamically stable. There was no concern for rupture or dissection upon discharge. The decision was made to conservatively manage with serial imaging and close observation. At the patient's subsequent 6-month follow up, a repeat CTA revealed a 15% reduction in the size of the PAA and conservative management continues to be the treatment plan for our patient (Fig. 2a, Fig. 2b). There were no complications or adverse outcomes in this case. Our work has been reported in line with the SCARE 2020 criteria [7].

## 3 Discussion

The majority of PAAs tend to be at the base of the main pulmonary artery. Idiopathic aneurysms of the main pulmonary artery are rare with a poorly understood pathogenesis primarily due to the limited number of cases. Deterling et al. reported that PAAs were discovered in approximately 1 of 14,000 autopsies [8]. PAAs are often diagnosed at autopsy, however an increased number of aneurysms are being discovered incidentally on imaging [[9], [10], [11], [12]]. The frequency of PAAs has been linked to several risk factors, including congenital heart abnormalities, pulmonary hypertension, trauma, atherosclerosis, infection, cystic medial necrosis, Bechet's disease, and collagen vascular diseases [6,13]. Our patient was diagnosed with respiratory syncytial virus 6 weeks prior to her emergency department visit, which could be a potential cause of her PAA, however this is unconfirmed.

PAAs are classified as acquired, congenital, and idiopathic [14]. A comprehensive review conducted by Gupta et al. found congenital causes comprised 25% of reported cases. Congenital heart defects lead to increased blood flow due to left-to-right shunting and promote aneurysm formation by increasing the shear stress on the vessel walls. Ventricular septal defects, patent ductus arteriosus, and atrial septal defects are considered the congenital heart defects most associated with PAAs [15]. Acquired causes of PAAs include untreated syphilis or tuberculosis, Marfan's syndrome, or pulmonary hypertension [16]. Historically, acquired causes resulted in the most PAAs. Lung cancer, autoimmune disease, and vasculitis (specifically Bechet's) have also been reported as potential causes [1,15]. Idiopathic is the least common classification of PAAs. Regardless of underlying etiology, pulmonary artery hypertension is the most common contributing factor to PAA formation. Symptoms of PAAs are dependent on the underlying etiology. The most common symptom is dyspnea, however for idiopathic PAAs, patients are typically asymptomatic [1]. If a patient with an idiopathic PAA becomes symptomatic, they may experience hemoptysis, chest pain, cough, palpitations, and dyspnea [16,17].

Management of PAAs is variable and generally depends on the underlying etiology, size of the aneurysm, and patient comorbidities [1]. Surgery is recommended when the patient is symptomatic and the diameter of the pulmonary artery is greater than 5 cm [18,19]. Common repair techniques include aneurysmorrhaphy for a focally dilated pulmonary artery, an allograft or synthetic graft for lesions caused by inflammation or pulmonary hypertension, or an interpositional graft for lesions involving the branches of the main pulmonary artery [16]. If a patient is experiencing severe hypertension, compression of neighboring anatomical structures, or rapid growth of the aneurysm, surgery is recommended to relieve symptoms and decrease the risk of fatal outcomes [16]. Surgical risk is greatly increased in patients with pulmonary hypertension [1]. Complications of surgery include artery dissection, rupture, thrombosis, and compression of the airway [18,19]. There are no clear guidelines for management of PAAs, primarily due to the severity of surgical complications. Choosing the least invasive approach is important because surgical techniques described previously are associated with a high morbidity and mortality risk [1].

Hemodynamic status and concurrent comorbidities play a role in treatment selection. Management of pulmonary hypertension and medical management of underlying disease is recommended for conservative treatment in asymptomatic and hemodynamically stable patients [1]. Routine radiographic follow-ups should also be performed to monitor the progression of the aneurysm [1]. It is important to monitor the patient at regular intervals and maintain follow up with CTA. The use of nonsurgical follow-up with serial CTA depends on the location, size, and stability of the PAA [3]. There are no current guidelines for diagnosis, management, or follow-up for these patients [1]. It is important to remember that PAA most commonly results secondary to underlying pathology, and each patient should have a treatment plan tailored to their unique circumstance.

## 4 Conclusion

Additional studies and cases are needed to develop more reliable approaches in managing patients with PAAs. This case highlights that conservative management should be considered first line therapy in asymptomatic, hemodynamically stable patients regardless of aneurysm size. Not all PAAs greater than 5 cm in diameter require immediate surgical intervention. Clinical judgment should be used to determine whether surgery is indicated as certain risk factors place individuals at higher risk for aneurysmal dissection, rupture, and death. However, in stable patients without risk factors, serial imaging should be considered first.

## Provenance and peer review

Not commissioned, externally peer-reviewed.

## Sources of funding

This research did not receive any specific grant from funding agencies in the public, commercial, or not-for-profit sectors.

## Ethical approval

Given the nature of the article, a case report, no ethical approval was required.

## Consent for publication

Written informed consent was obtained from the patient for publication of this case report and accompanying images. A copy of the written consent is available for review by the Editor-in-Chief of this journal on request.

## Author contributions

Dr. Hafez Golzarian was involved in study concept, drafting, literature review, and editing of the manuscript.

Dr. Sreenivasa Chanamolu was responsible for revising the manuscript for important intellectual content and supervising.

Morgan Turnow was responsible for study concept, drafting, literature review, and revising the manuscript.

Sidney Elston was responsible for study concept, drafting, literature review, and revising the manuscript.

## Research registration

This is not an original research project involving human participants in an interventional or an observational study but a case report. This registration was not required.

## Guarantor

Dr. Hafez Golzarian, DO.

## Funding

This study did not receive any funding relative to its elaboration. Conflicts of Interest: None. Ethical approval and informed consent (to participate and for publication): Completed. Availability of data and material (data transparency): No applicable to this study. Author contributions: Conception and design of study: all authors; Acquisition of data: all authors; Analysis and/or interpretation of data: all authors; Drafting the manuscript: all authors; Revising the manuscript critically for important intellectual content: all authors; Approval of the version of the manuscript to be published: all authors.

## Declaration of competing interest

The authors have no conflict of interest to declare.
